# Short term effects of anodal cerebellar vs. anodal cerebral transcranial direct current stimulation in stroke patients, a randomized control trial

**DOI:** 10.3389/fnins.2022.1035558

**Published:** 2022-11-24

**Authors:** Qurat-ul-ain, Zafran Ahmad, Summaiya Ishtiaq, Saad Ilyas, Irum Shahid, Iqbal Tariq, Arshad Nawaz Malik, Tian Liu, Jue Wang

**Affiliations:** ^1^The Key Laboratory of Biomedical Information Engineering of Ministry of Education, Institute of Health and Rehabilitation Science, School of Life Science and Technology, Xi’an Jiaotong University, Xi’an, China; ^2^National Engineering Research Center for Healthcare Devices Guangzhou, Guangzhou, Guangdong, China; ^3^The Key Laboratory of Neuro-informatics & Rehabilitation Engineering of Ministry of Civil Affairs Xi’an, Xi’an, Shaanxi, China; ^4^School of Economics and Management, Yunnan University, Kunming, China; ^5^Department of Rehabilitation Sciences, Shifa Tameer-e-Millat University, Islamabad, Pakistan; ^6^Faculty of Computing, Capital University of Science and Technology, Islamabad, Pakistan; ^7^Institute of Physical Medical and Rehabilitation, Khyber Medical University, Peshawar, Pakistan; ^8^Faculty of Rehabilitation and Allied Health Sciences, Riphah College of Rehabilitation and Allied Health Sciences, Islamabad, Pakistan

**Keywords:** balance, cerebellar transcranial direct current stimulation, cerebral transcranial direct current stimulation, cognition, gait, stroke

## Abstract

**Background:**

Balance and gait impairments are major motor deficits in stroke patients that require intensive neuro-rehabilitation. Anodal transcranial direct current stimulation is a neuro-modulatory technique recently used in stroke patients for balance and gait improvement. Majority of studies focusing on tDCS have assessed its effects on cerebral motor cortex and more recently cerebellum as well but to our best knowledge the comparison of stimulating these two regions in stroke patients is not investigated so far.

**Objective:**

The current study aimed to compare the effect of anodal transcranial direct current stimulation on cerebellar and cerebral motor cortex M1 in stroke patients.

**Materials and methods:**

This double-blinded, parallel, randomized, sham controlled trial included 66 patients with a first-ever ischemic stroke were recruited into three groups; Cerebellar stimulation group (CbSG), M1 Stimulation Group (MSG), and Sham stimulation group (SSG). A total of three sessions of anodal transcranial direct current stimulation were given on consecutive days in addition to non-immersive virtual reality using Xbox 360 with kinect. Anodal tDCS with an intensity of 2 mA was applied for a duration of 20 min. Primary outcome measures berg balance scale (BBS), timed up and go test (TUG), BESTest Balance Evaluation–Systems Test (BESTest) and secondary outcomes measures montreal cognitive assessment (MoCA), mini mental state examination (MMSE), Johns Hopkins Fall Risk Assessment Tool (JHFRAT), twenty five feet walk test (25FWT), six minute walk test (6MWT), and tDCS Adverse Effects was assessed before initiation of treatment (T0) and at the end of third session of stimulation (T1).

**Results:**

The results of between group’s analysis using mean difference showed a significant difference with *p*-value <0.05 for balance (BBS, TUG, BESTest), walking ability (6MWT, 25FWT), risk of fall (JHFRAT). Cognitive function did not show any significant change among the groups for MoCA with *p*-value >0.05 but MMSE was improved having significant *p*-value (*p* = 0.013). However, 6MWT and 25FWT showed non-significant results for both between group and within group analysis. In pairwise comparison both the cerebellar and cerebral stimulation groups showed Significant difference with *p*-value <0.05 in comparison to sham stimulation; BBS (cerebellar vs. sham *p* ≤ 0.001, cerebral vs. sham *p* = 0.011), TUG (cerebellar vs. sham *p* = 0.001, cerebral vs. sham *p* = 0.041), Bestest (cerebellar vs. sham *p* = 0.007, cerebral vs. sham *p* = 0.003). Whereas for JHFRAT only cerebellar stimulation in comparison to sham and motor cortex stimulation showed significant improvements (cerebellar vs. M1 *p* = 0.037, cerebellar vs. sham *p* = 0.037). MMSE showed significant improvement in M1 stimulation (M1 vs. cerebellar *p* = 0.036, M1 vs. sham *p* = 0.011).

**Conclusion:**

Findings of the study suggest anodal tDCS stimulation of the cerebellum and cerebral motor cortex both improves gait, balance and risk of fall in stroke patients. However, both stimulation sites do not induce any notable improvement in cognitive function. Effects of both stimulation sites have similar effects on mobility in stroke patients.

## Introduction

Stroke frequently leads to incomplete motor recovery and hence stroke is the third leading root cause of global disability (Norrving and Mensah, 2017). One of the major cause of disability after a stroke is motor impairment, which limits the level of function and mobility (Kim et al., 2014). Following a stroke, people commonly experience gait impairments, which hinder their ability to carry out daily tasks and greatly impact their quality of life (Robinson et al., 2011). As a matter of fact, post-stroke gait rehabilitation plans generally focus on improving general walking abilities, gait speed, walking cadence, and lower limb (LL) muscle strength (Richards et al., 1999; Langhorne et al., 2009; Xu et al., 2015; Vaz et al., 2019). Stroke related neuro-rehabilitation has been improved by techniques that promote the brain’s capacity to rebalance the excitability of both hemispheres (Stoykov and Madhavan, 2015). Tran-scranial direct current stimulation (tDCS) is a priming technique that modulates membrane potential and initiates synaptic plasticity in the area of application as well as the adjacent neural networks (Stagg and Nitsche, 2011). TDCS stimulation in addition to therapies including conventional rehabilitation, robotics based rehabilitation, virtual reality, physical therapy and other techniques is a promising intervention to enhance motor performance, balance and gait in stroke patients (Geroin et al., 2011; Seo et al., 2017; Picelli et al., 2018; Ehsani et al., 2022; Salameh et al., 2022).

Over the last few decades tDCS has been acknowledged as a very important neuro-modulatory technique in neuro-rehabilitation (Nitsche and Paulus, 2000; Nitsche and Paulus, 2001; Ehsani et al., 2016). The effects of tDCS are reliant on polarity, usage of a positive electrode over the aimed section of the brain i.e., anodal tDCS can potentially enhance cortical excitability, whereas the reverse or opposite effects can be obtained by application of a negative electrode i.e., cathodal tDCS (Nitsche and Paulus, 2000; Roche et al., 2015). According to the literature, different cortical regions such as the primary motor cortex M1, supplementary motor cortex and pre motor cortex, basal ganglia, and cerebellum are all included in a network that contributes to motor skill acquirement during motor learning (Doya, 2000; Krings et al., 2000; Ungerleider et al., 2002). Some studies in this field have found that a-tDCS of primary motor cortex improves both motor learning and motor function (Hummel et al., 2005; Vines et al., 2008; Reis et al., 2009; Sohn et al., 2013; Manji et al., 2018). Literature also confirms that Error-based motor learning, also known as motor adaptation, has been linked to the cerebellum (Caligiore et al., 2017). Purkinje cells’ long-term depression-like plasticity is linked to learning; this Hebbian process is triggered by the activation of both climbing and parallel fibers simultaneously, which provide input to the cortex in the form of error messages in motor control (Jayaram et al., 2011; Ito et al., 2014). Hence, the cerebellar hemispheres specifically play a role in motor adaptation (Galea et al., 2011), and balance performance can be considered as a sort of postural adaptation (Morton and Bastian, 2004). More precise movement endpoints are also a result of a robust motor cortex-cerebellar interaction, highlighting the critical function of the cerebellum in motor adaptation (Schlerf et al., 2015; Caligiore et al., 2017). Although the cerebellum’s more medial flocculonodular lobe is specifically related to postural balance, its anterior placement makes it unlikely that it can be targeted with tDCS, whereas the hemispheres can (Rampersad et al., 2014). Numerous studies have shown that anodal stimulation of cerebellum significantly enhances motor adaptation and balance performance (Galea et al., 2009, 2011; Ferrucci et al., 2013; Hardwick and Celnik, 2014; Foerster et al., 2017; Poortvliet et al., 2018; Liebrand et al., 2020).

Although the number of studies focusing on tDCS stimulation in stroke patients mainly focuses on upper limb function yet there are several studies focusing on LL functions. For LL M1 stimulation the targeted tDCS application is difficult as cortex area for LL is more medial and deeper within the inter-hemispheric fissure than upper limb (Gowan and Hordacre, 2020). Evidence suggests that anodal tDCS (2 mA) can be used to modulate activity in the LL M1 (Jeffery et al., 2007). In 2013 Sohn et al. found anodal tDCS to be effective in improving balance and lower extremity strength in stroke patients (Sohn et al., 2013). Different montages of tDCS stimulation are also found to be reducing risk of falls and improving LL function in post-stroke patients (Andrade et al., 2017). A recent systematic review published in 2021 states also declares M1 tDCS stimulation to be effective in enhancing stroke rehabilitation by improving LL function, gait parameters and both static as well as dynamic balance (Navarro-López et al., 2021).

Despite the fact that M1 has received attention as a target stimulation site to alter the excitability of the motor cortex for the LL (M1), cerebellar tDCS also has the potential to induce comparable neuro-physiological changes (Rampersad et al., 2014). A computational modeling study comparing electrode montages designed to target M1 and the cerebellum discovered that stimulating the cerebellum can generate significantly higher electric field strengths in the stimulated region than Motor cortex stimulation, implying that the cerebellum could indeed be a prime target for tDCS (Rampersad et al., 2014; Gowan and Hordacre, 2020). Research evidence greatly lacks regarding effect of cerebellar stimulation in improving LL, gait or balance function in stroke patients yet some researchers found it to be an effective intervention. Rezaee et al. in 2020 found cerebellar tDCS to be promising intervention for enhancing functional reach while maintaining standing balance in chronic stroke (Rezaee et al., 2021). Another study also support short term effects of anodal tDCS for improving standing balance performance in stroke patients compared to healthy adults (Zandvliet et al., 2018). Recently, Solanki and colleagues found single session of cerebellar stimulation to be effective in inducing immediate enhancement of gait performance and balance in stroke patients (Solanki et al., 2021). Similarly Mohammadi and colleagues reported an enhancement of balance function in stroke patients after a single session of cerebellar stimulation (Mohammadi et al., 2021).

Although several studies suggested the promising results of stimulating motor cortex and cerebellum in stroke patients yet hardly any study compared the effect of tDCS stimulation on both target areas. Therefore current study was designed to investigate if short term tDCS stimulation of motor cortex and cerebellum has any additional benefits over each other in comparison to sham stimulation.

## Materials and methods

A double-blind randomized sham-controlled clinical trial was conducted in order to investigate the short-term effects of brain stimulation using anodal tDCS. The study was conducted at Akbar Hospital, Gujrat, Pakistan. All the procedures were performed adhering to the Declaration of Helsinki. Research Protocol of the study was approved by the ethics committee of Riphah College of Rehabilitation Sciences Riphah International University, Islamabad “Research Ethical Committee” on October 10, 2021 (Ref: RIPHAH/RCRS/REC/Letter/01142). Informed signed consent was obtained by all participants prior to inclusion in the study and all procedures along with risk factors were well explained to the participants. The study was registered under the U.S. National Library of medicine within ClinicalTrials.gov (clinicaltrials.gov) (NCT05115851) on November 10, 2021. The Sample size of the study was calculated using G power 3.1 (Faul et al., 2007; Yosephi et al., 2018) (Heinrich-Heine-Universität Düsseldorf, Düsseldorf, Germany) and data collection process was ended once the required sample size was obtain excluding all the dropouts. The participants of the study were free to quit the trial at any time on their own choice.

## Subjects

**FIGURE 1:**
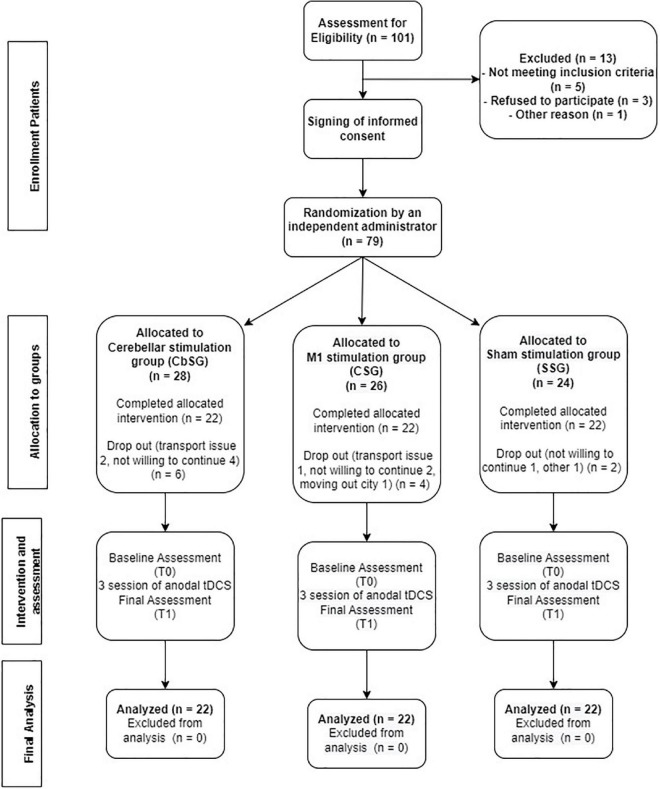
CONSORT flowchart diagram.

A total number of 105 participants were initially assessed to participate in the study and 66 participants completed the study after recruitment and dropouts (Figure 1). Stroke patients having first ever stroke attack of either gender, age ranging from 40 to 80 years, and scoring six or above on Johns Hopkins Fall Risk Assessment Tool (JHFRAT) were recruited in the study. Participants included must be capable to provide written consent, walking unassisted, and having a functional status that allows them to actively engage in exercise therapy on the Xbox training program. Initial assessment excluded all participants having any co-morbid neurological condition including Alzheimer, Parkinson, Cerebellar disorder, Psychological illnesses, having score below 21 on Mini Mental Status Examination Test (MMSE), signs of motor disorder affecting gait or LL function, sedative medicines, Amnesia, Depression, radiculopathy or lumbar spinal cord root involvement, any auditory/visual impairment, Vertigo, having recent fracture, severe cardiac issues and recipients of electrotherapy that may have affected the nervous system in the 2 weeks prior to the study.

### Randomization and blinding procedure

Central randomization was conducted using computer-generated randomization service^1^ and patient allocation was effectuated by an independent administrator. The interventions were performed by trained physical therapist and the data was collected by independent assessor. Therapists, assessors, and participants were blinded to group allocation throughout the duration of the trial. Patient identification was kept confidential and any personal information that may identify participant’s identity was not a requirement of the study. Confidentiality of patient information was maintained by the assessor and investigators.

### Study procedures and intervention

The participants of the current sham-controlled study were divided into two interventional and a sham group. A total of 66 participants were equally enrolled in each of the three groups. The groups included Cerebellar Stimulation Group (CbSG), M1 Stimulation Group (MSG), and Sham Stimulation Group (SSG). All participants received three session of anodal transcranial direct current stimulation along with non-immersive Virtual Reality based LL functional training using Xbox 360 with kinect. Blind assessor collected data using a semi-structured questionnaire and outcome measure tools: Berg Balance Scale (BBS), Timed Up and Go Test (TUG), Six Minute Walk Test (6MWT), 25 Feet Walk Test (25FWT), Johns Hopkins Fall Risk Assessment Tool (JHFRA), BESTest Balance Evaluation–Systems Test Post (BESTest) along with six Components, Mini-Mental State Examination (MMSE), Montreal Cognitive Assessment (MoCA), and tDCS Adverse Effects Questionnaire (Brunoni et al., 2011). Baseline assessment was carried out prior to first session of intervention (T0) and after completion of all three sessions of anodal tDCS stimulation and Xbox Kinect training (T1). Data from patients who completed trial successfully was included in the study.

### Transcranial direct current stimulation

**FIGURE 2:**
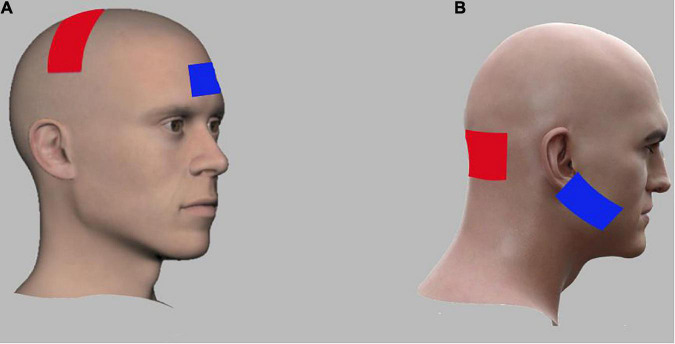
Electrode montages for anodal tran-scranial direct current stimulation (tDCS) of M1 and cerebellar stimulation groups. **(A)** For M1, the anodal electrode (red) was placed over the lesioned M1 region while the cathodal electrode (blue) was placed over the right contralateral supraorbital region. **(B)** For Cerebellum, the anodal electrode (red) was placed over the cerebellum bilaterally (1 cm below inion of occiputal bone) while the cathodal electrode (blue) was placed over the right buccinators muscle.

Anodal transcranial direct current stimulation was given using a portable battery-driven brain stimulator (The Brain Stimulator v3.0 Deluxe tDCS Kit, using Professional 3 x 3 inches Amrex Sponge Electrodes). Skin was prepared before application of electrodes by cleaning the skin surface using alcohol swabs. In cerebellar stimulation group (CbSG) a stimulation intensity of 2 mA for a duration of 20 min was applied. In order to avoid sudden initiation and termination of the stimulation (Brunoni et al., 2011) the first and last 10 s of anodal tDCS application, current will be gradually fade in/fade out (Nitsche and Paulus, 2000; Samaei et al., 2017) to avoid any sudden starting or stopping of the stimulation. For the CbSG active anodal electrode was placed over the cerebellum about 1–2 cm below inion occipital protuberance, whereas the returning cathodal electrode was placed on right buccinator muscle as shown in Figure 2B (Zuchowski et al., 2014; Yosephi et al., 2018).

In the M1 Stimulation Group (MSG) a similar intensity of current with 2 mA was applied for a period of 20 min. Skin was cleaned with alcohol swab ahead of placing the electrodes. Anode was positioned over the lesioned M1 motor cortex area (C3, International 10–20 system) while the cathode was placed over the contra-lateral supraorbital area as shown in Figure 2A. Due to the large size of electrodes, the active electrode covered the area of M1 for the hand, arm, trunk, and the LL (Nitsche et al., 2003; Gandiga et al., 2006; Yosephi et al., 2018). Three consecutive sessions of anodal tDCS were applied for 3 days along with virtual reality based training.

In Sham Stimulation group (SSG) three consecutive sessions of anodal tDCS of same intensity i.e., 2 mA stimulation was applied for a period of 30 s and then gradually ramped-down and turned off for the rest of the treatment time of 20 min. Same procedure for skin preparation was used as in other two groups. Electrode placement for SSG was same as in MSG; anode will be placed over the left (dominant) lesioned M1 whereas the cathode will be positioned over the right (contralateral) supraorbital area (Nitsche et al., 2003; Gandiga et al., 2006; Yosephi et al., 2018).

### Xbox 360 Kinect Training

Balance and mobility training for rehabilitation of LL function was carried out by exer-gaming using Xbox Kinect 360 for duration of 50 min along with brain stimulation. The equipment included LED screen, an infrared camera/Kinect sensor to recognize participant’s movement and console. The gaming system was installed in a dedicated room to avoid patient distraction. Participants stood approximately 1.5–2 m from the monitor (Luna-Oliva et al., 2013; Jung et al., 2021). Games that were subjected to be played by the individual included those specified for LL i.e., soccer; beach volleyball and basketball in Kinect Sports: Season 1.16. All three games; Soccer, Beach Volleyball, and Basketball involve active movements on hip, knee, and ankle. Hip active movements; hip flexion, extension, abduction, adduction, external rotation, and internal rotation. Active movements at knee included flexion and extension. Active movement on ankle; ankle dorsiflexion and plantar flexion (Jung et al., 2018). Physiotherapists demonstrated participants from all three groups during the first week, which served as an orientation week. All three games were played for nearly 15 min with a rest time of 2–5 min.

### Statistical analysis

All statistical analysis of the sample were performed using IBM SPSS 20 by the principal investigator. Data normality was assessed using Shapiro-Wilk Test for all primary and secondary tools and is represented in Table 1. Based on the Shapiro-Wilk test Montreal Cognitive Assessment Score, component four and six of BESTtest including BESTest Reactive Postural Response and BESTest Stability in Gait were normally distributed so One Way ANOVA with *post-hoc* test was used to analyze these variables. All other variables that were not normally distributed were analyzed using non-parametric Kruskal–Wallis test and Bonferroni’s method was used for pair wise multiple comparisons. BESTest tool has six sub-components that were also analyzed individually as a variable. Values of *P* < 0.05 indicated statistically significant differences.

**TABLE 1:** Test of normality (Shapiro-Wilk test) for all outcome tools.

Variable	Shapiro-Wilk test
Berg Balance Scale	0.001
Timed Up and Go Test	<0.001
Six Minute Walk Test	<0.001
25 Feet Walk Test	<0.001
Johns Hopkins Fall Risk Assessment Tool	<0.001
BESTest Balance Evaluation – Systems Test	0.047
BESTest Biomechanical Constraints	0.002
BESTest Stability Limits	<0.001
BESTest Transitions-Anticipatory Postural Adjustment	0.016
BESTest Reactive Postural Response	0.053
BESTest Sensory Orientation	<0.001
BESTest Stability In Gait	0.071
Mini-Mental State Examination	0.001
Montreal Cognitive Assessment	0.066

## Results

The results were derived by the final analysis of 66 participants who completed the study. Mean age of the participants in all three groups were to some extent similar. The demographic characteristics of the three groups are compared in Table 2.

**TABLE 2:** Demographic characteristics of study population.

Variable	Cerebellar stimulation group (CbSG) (*n* = 22)	M1 stimulation group (MSG) (*n* = 22)	Sham stimulation group (SSG) (*n* = 22)
Age (Mean ± SD)	56.18 ± 6.3	57.91 ± 5.75	58.36 ± 5.9
Gender (Male, Female)	16, 6	20, 2	18, 4
Stroke type (Ischemic, Hemorrhagic)	16, 6	18, 4	16, 6
Hemisphere affected (Right, Left)	10, 12	12, 10	11, 11
Dominant hemisphere (Right, Left)	0, 22	2, 20	0, 22
Ashworth (Mean ± SD)	0.82 ± 0.7	1.18 ± 0.7	0.64 ± 0.5
Duration (Mean ± SD)	17.63 ± 13.75	10.45 ± 9.3	18.18 ± 13.1

The baseline score of all the variables tested are mentioned in Table 3 along with the *p*-value calculated by ANOVA. The Shapiro-Wilk value given in Table 1 and the *p*-value clearly indicate the data is non-homogenous so the test used for analysis of these variables were chosen accordingly as mentioned in statistical analysis.

**TABLE 3:** Baseline scores (mean standard ± deviation) for all variables.

Variable	Cerebellar stimulation group (CbSG) *n* = 22 (Mean ± SD)	M1 stimulation group (MSG) *n* = 22 (Mean ± SD)	Sham stimulation group (SSG) *n* = 22 (Mean ± SD)	Homogeneity of variances (*P*-value)
Berg Balance Scale	41.63 ± 8.2	40.27 ± 8.4	50.27 ± 3.1	<0.001
Timed Up and Go Test	17.54 ± 7.6	13.85 ± 4.9	9.69 ± 2.6	<0.001
Six Minute Walk Test	0.12 ± 0.03	0.13 ± 0.03	0.17 ± 0.8	0.006
25 Feet Walk Test	18.47 ± 12.8	14.07 ± 6.8	10.60 ± 7.0	0.025
Johns Hopkins Fall Risk Assessment Tool	9.81 ± 4.9	12.82 ± 3.5	10.00 ± 2.8	0.02
BESTest Balance Evaluation – Systems Test	62.72 ± 15.26	59.27 ± 17.8	75.18 ± 10.1	0.002
BESTest Biomechanical Constraints	8.45 ± 2.5	7.54 ± 3.9	10.00 ± 2.3	0.029
BESTest Stability Limits	13.54 ± 3.9	14.09 ± 3.5	15.36 ± 1.9	0.169
BESTest Transitions-Anticipatory Postural Adjustment	8.36 ± 2.3	8.54 ± 2.9	11.54 ± 2.8	<0.001
BESTest Reactive Postural Response	8.81 ± 2.3	7.18 ± 3.2	9.90 ± 2.7	0.007
BESTest Sensory Orientation	12.73 ± 2.3	12.64 ± 2.2	14.00 ± 0.9	0.034
BESTest Stability In Gait	9.64 ± 5.8	9.45 ± 4.5	14.36 ± 2.5	0.001
Mini-Mental State Examination	22.81 ± 4.5	20.72 ± 5.0	23.45 ± 5.7	0.186
Montreal Cognitive Assessment	17.72 ± 4.88	18.09 ± 4.7	18.72 ± 4.0	0.764

**FIGURE 3:**
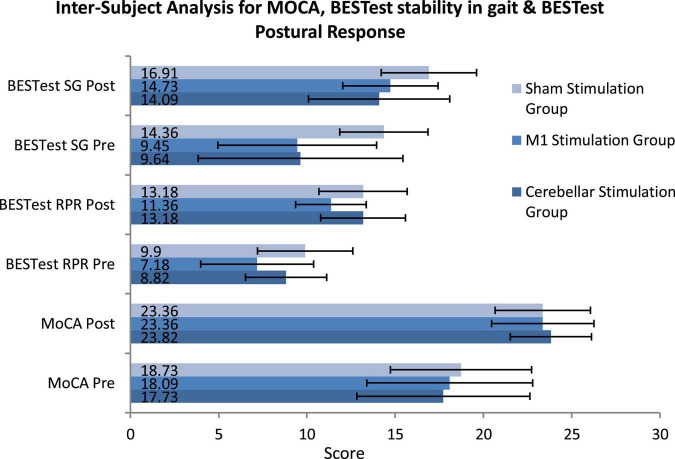
Inter-subject analysis for montreal cognitive assessment (MOCA), BESTest stability in gait and BESTest Postural Response using Mixed/Split Plot ANOVA (SG, stability in gait; RPR, reactive postural response; MoCA, montreal cognitive assessment).

Analysis across groups for normally distributed variables (MoCA, BESTest subgroup four Reactive Postural Response and six Stability in Gait) was conducted using mixed ANOVA/Split Plot ANOVA. The *P*-value for between subject factors for MoCA did not showed significant result with *p*-value >0.05 (*p* = 0.941, *df* = 2) where as the other two subgroups of BESTest four (*p* = 0.008*, *df* = 2) and six (*p* = 0.001**, *df* = 2) showed Significant results as presented in Figure 3 (Supplementary Table 1). Mean Difference was calculated using compute variable feature on SPSS and analyzed by applying one-way ANOVA Mean difference for MoCA was non-significant (*p* = 0.396, *df* = 2, η _*p*_^2^ = 0.029), however subgroups four reactive postural control (*p* = 0.041*, *df* = 2, η _*p*_^2^ = 0.096) and subgroup six stability in gait (*p* = 0.045, *df* = 2, η _*p*_^2^ = 0.094) were statistically significant.

**FIGURE 4:**
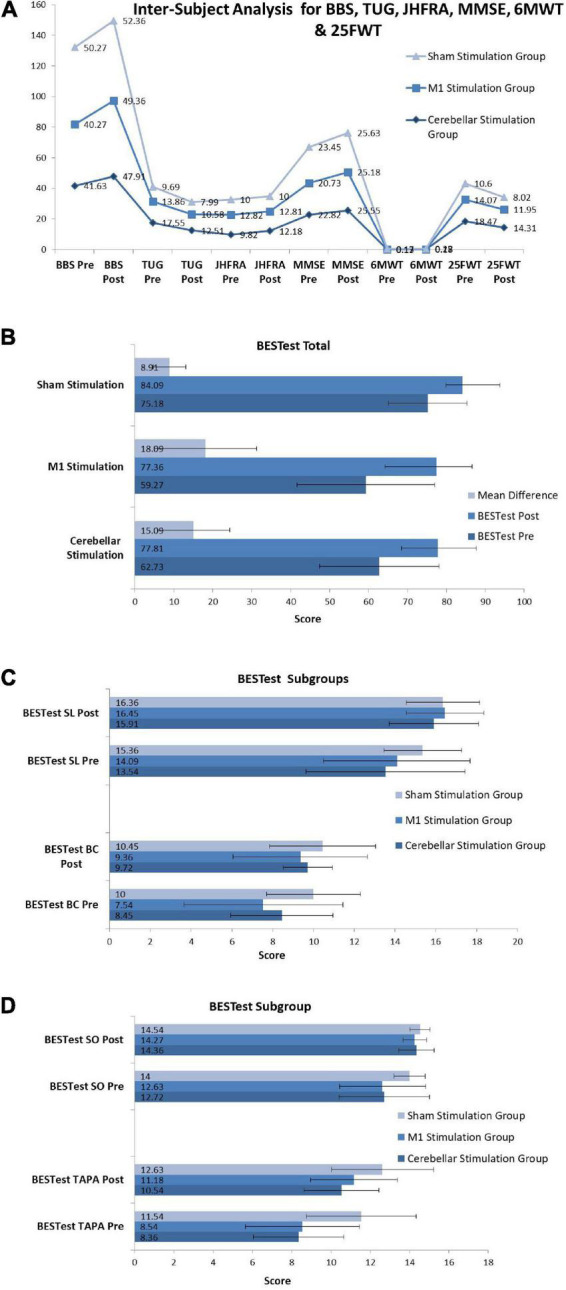
Inter-subject analysis for **(A)** BBS (Berg Balance Scale), TUG (Timed Up and Go Test), JHFRAT (Johns Hopkins Fall Risk Assessment Tool), MMSE (Mini-Mental State Examination), 6MWT (Six Minute Walk Test) and 25FWT (Twenty Five Feet Walk Test). **(B)** BESTest (BESTest Balance Evaluation–Systems Test). **(C)** BESTest sub-groups SL and BC (Stability Limits, Biomechanical Constraints). **(D)** BESTest sub-groups SO and TAPA (Sensory Orientation, Transitions-Anticipatory Postural Adjustment).

The Kruskal Wallis H Test was used for across-group analysis for all skewed variables, and the results are presented in Figure 4. Due to the skewed nature of the data most of the variables already showed significant *p*-values at baseline as well as after intervention (Supplementary Table 2) so we have calculated the mean difference for all variables in each group to find the mean difference changed after three sessions of intervention. The mean difference was also analyzed by Kruskal Wallis H Test and indicates a statistically significant change for BBS (*p* = 0.001**, *df* = 2, H = 14.10, η _*p*_^2^ = 0.200), TUG (*p* = 0.004*, *df* = 2, H = 11.01, η _*p*_^2^ = 0.163), JHFRAT (*p* = 0.015*, *df* = 2, H = 8.39, η _*p*_^2^ = 0.129), BESTest total score (*p* = 0.004*, *df* = 2, H = 10.85, η _*p*_^2^ = 0.142), BESTest subgroup one biomechanical constraints (*p* = 0.034*, *df* = 2, H = 6.79, η _*p*_^2^ = 0.092) and three transitions-anticipatory postural (*p* = 0.004*, *df* = 2, H = 11.15, η _*p*_^2^ = 0.153) and MMSE (*p* = 0.027*, *df* = 2, H = 7.25, η _*p*_^2^ = 0.128). Non-significant results were found with *p*-value > 0.05 for 6MWT (*p* = 0.435, *df* = 2, H = 1.66, η _*p*_^2^ = 0.048), 25FWT (*p* = 0.141, *df* = 2, H = 3.92, η _*p*_^2^ = 0.039), BESTest subgroup two stability limits (*p* = 0.215, *df* = 2, H = 3.07, η _*p*_^2^ = 0.087) and five sensory orientation (*p* = 0.074, *df* = 2, H = 5.19, η _*p*_^2^ = 0.08). Value of H was calculated using formula

H=′Σn⁢(n+1)12(njR2j)-3(n+1)

$$H={}_{1-\text{correction}}^{H^{\prime }}$$

Where R_*j*_ denotes the rank sum of group j, n_*j*_ denotes the sample size of group j and n denotes the total sample size across all groups, n = n_1_ +…+ n_*j*_.

**FIGURE 5:**
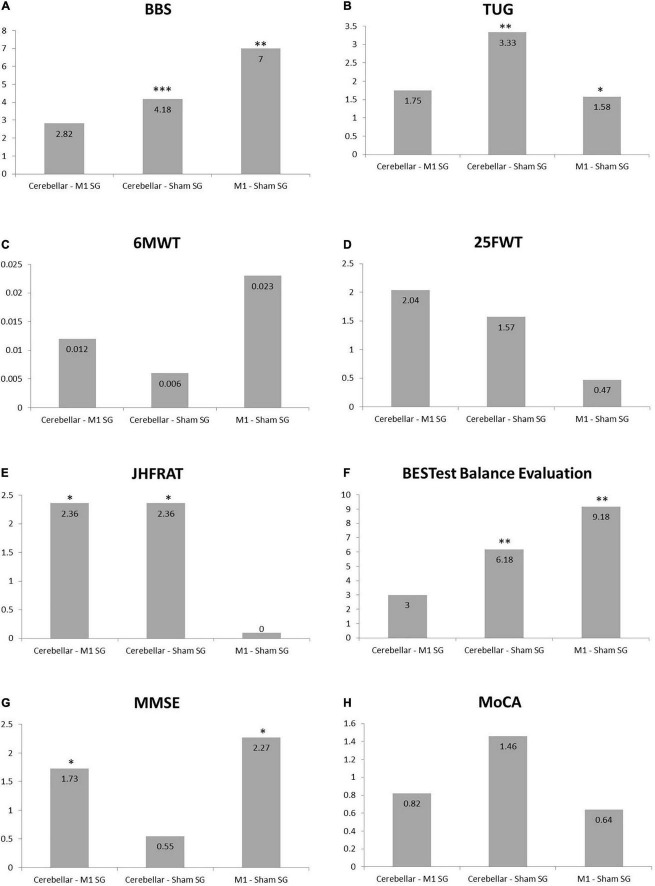
Bar charts showing Bonferroni’s method for pair wise multiple comparisons. **(A)** Berg Balance Scale. **(B)** Timed Up and Go Test. **(C)** Six Minute Walk Test. **(D)** Twenty Five Feet Walk Test. **(E)** Johns Hopkins Fall Risk Assessment Tool. **(F)** BESTest Balance Evaluation–Systems Test. **(G)** Mini-Mental State Examination. **(H)** Montreal Cognitive Assessment, *indicates significance difference among the pair of groups. *P ≤ 0.05, **P ≤ 0.01, and ***P ≤ 0.001.

Multiple group analysis was carried out to further clarify the significant effect of each group in comparison to the other two groups using Bonferroni’s method for pair wise comparison using mean difference scores as shown in Figure 5. For berg balance scale and timed up and go test significant difference was found for both cerebral stimulation and cerebellar stimulation in comparison to sham stimulation. However, all pairs of the interventional groups revealed non-significant results for the 6MWT and 25FWT. Fall of risk assessed by JHFRAT did not changed from pre- to post-scores for cerebral stimulation and sham stimulations groups as it can be seen in Figure 5, therefore JHFRAT was significantly improved (*p* = 0.037) in cerebellar stimulation group in comparison to other two stimulation groups. For BESTest total score and most of its components similar results were found like BBS and TUG that were significantly improved for both stimulation sites in comparison to sham stimulation. Subgroup biomechanical constraints and stability in gait showed significant improvement only in motor cortex stimulation in comparison to sham stimulation. Regarding cognitive function MoCA scores remained non-significant in all pairs where as MMSE showed significant improvement in motor cortex stimulation group in comparison to both cerebellar stimulation and sham stimulation with *p*-value < 0.05 (*p* = 0.036, *p* = 0.011). Details for pair wise comparison (mean difference, 95% CI, *P*-value) are given in Supplementary Table 3.

Transcranial direct current stimulation was well tolerated by all of the three interventional groups with minimal side effects or adverse effects, however 72.7% participants in the cerebellar stimulation group reported headache. Table 4 reports mean and standard deviation for all abnormal sensations among three groups. Only four abnormal sensations were reported i.e., headache, tingling, itching, and skin redness. The scale was scored from 1 to 4 with one: absent, two: mild, three: moderate, four: severe and five was scored in case if patient is definite about the cause of symptom being tDCS stimulation. In CbSG six participants score one (absent), four participants score four (severe) and 12 participants scored five (definite) for headache; four participants reported five (definite) for tingling; none reported itching and two reported five (definite) for skin redness. In MSG only two participants reported four (severe) for headache; two (mild), four (severe), five (definite) were each reported by two participants for tingling; four (severe), five (definite) were each reported by two participants for itching; and none reported skin redness. The least number of sensations were reported for sham stimulation group where only two participants were definite (five) for both tingling and itching. Participants, on the other hand, reported no burning sensations or pain during stimulation. Furthermore, no abnormal sensations were reported by the participants following stimulation.

**TABLE 4:** Mean score with SD for adverse effects of transcranial direct current stimulation (tDCS) among all groups.

Sensation	Cerebellar stimulation group CbSG (Mean ± SD)	M1 stimulation group (MSG) (Mean ± SD)	Sham stimulation group SSG (Mean ± SD)
Headache	3.73 ± 1.8	1.27 ± 0.9	1.00 ± 0
Neck pain	1.00 ± 0	1.00 ± 0	1.00 ± 0
Scalp pain	1.00 ± 0	1.00 ± 0	1.00 ± 0
Tingling	1.73 ± 1.6	1.73 ± 1.4	1.36 ± 1.2
Itching	1.00 ± 0	1.64 ± 1.4	1.36 ± 1.2
Burning sensation	1.00 ± 0	1.00 ± 0	1.00 ± 0
Skin redness	1.36 ± 1.2	1.00 ± 0	1.00 ± 0
Sleepiness	1.00 ± 0	1.00 ± 0	1.00 ± 0
Trouble concentrating	1.00 ± 0	1.00 ± 0	1.00 ± 0
Acute mood change	1.00 ± 0	1.00 ± 0	1.00 ± 0
Others	1.00 ± 0	1.00 ± 0	1.00 ± 0

## Discussion

To the best of our knowledge current study is the first of its kind to compare the short term effects of anodal tDCS on motor cortex M1, cerebellum and sham stimulation in stroke patients. The results of the current study indicate that short term anodal tDCS stimulation is effective in improve lower limb and balance outcomes in stroke patients. Both motor cortex stimulation and cerebellum stimulation improved balance (BBS, TUG, BESTest) in comparison to sham stimulation. Mobility and endurance (6MWT, 25FWT) was not improved in any of the group by short term stimulation, however risk of fall was reduced in cerebellar stimulation group. Cognitive function can be enhanced by cerebral stimulation.

The findings of the study that cerebral M1 stimulation or cerebellar stimulation significantly enhances the effect of balance and gait training are supported by many studies (Vines et al., 2008; Zandvliet et al., 2018; Mohammadi et al., 2021; Hummel et al., 2005). Similar to our study Craig and Doumas in 2017 also investigated effect of tDCS on both M1 and cerebellum; they suggested enhancing effect of motor cortex and cerebellar anodal tDCS stimulation on balance (berg balance scale) in both young and older population. But, this study in contrast to our study did not compared the effect of cerebellar versus motor cortex stimulation (Craig and Doumas, 2017). Another similar study by researchers Yosephi et al. evaluated the effects of postural training with cerebellar anodal tDCS, motor cortex anodal tDCS, and sham stimulation on balance and postural stability. In contrast to our study, their population was an aged population with a higher risk of falls rather than stroke. They discovered that while both therapies (M1 and cerebellar tDCS) could considerably improve stability and balance, cerebellar stimulation was more effective than motor cortex stimulation in improving dynamic postural control. However in the current study, we found both motor cortex and cerebellar stimulation to be equally effective, and postural sub-components of Bestest balance evaluation system also showed equal effectiveness of both stimulations in comparison to sham stimulation (Yosephi et al., 2018). Regarding balance function, our results were in line with another study recently conducted that compared the effects of anodal tDCS on the cerebellum and motor cortex again in the geriatric population rather than stroke that both therapies had a similar impact (Baharlouei et al., 2020).

Both motor cortex and cerebellum have strong physiological basis to be capable of enhancing balance and motor function. The cerebellum governs the muscles used for balance by receiving and processing information from multiple systems including somato-sensory, vestibular, auditory and, visual system (MacLullich et al., 2004; Paul et al., 2009). The vermis is crucial for balance, and cerebellar white matter tracts link the cerebellum to other parts of the brain (Shumway-Cook and Woollacott, 2007). According to existing evidence, a-tDCS can improve postural control by altering the intricate linkages between the motor cortex and cerebellum and by manipulating how the vermis works. Additionally, tDCS can enhance Purkinje cell activation and hence improve the performance of both white matter tracts and vermis (Celnik, 2015). Moreover, the motor cortex is a component of the cortico-basal ganglia network, which is vital in balance control (MacLullich et al., 2004). Numerous investigations demonstrated that tDCS could affect cerebral cortical activity (Kim et al., 2012), the corticospinal tract, and the excitability of the spinal network (Roche et al., 2009). Neuronal excitability in the cortical network is enhanced by anodal tDCS and that balancing tasks boosted synaptic activity, resulting in better balance indices (Kaski et al., 2012). Based on the physiological considerations it is difficult to determine which of the two stimulation sites can be more beneficial, thus it is the need of time to undertake trials with a variety of populations and traits to clarify the therapeutic implications of stimulating the two sites to improve balance and lower limb function.

In the current study we applied anodal tDCS during online motor activity i.e., during gait and balance training on virtual reality based gait system. A study conducted by F. Ehsani et al. in 2016 concluded that anodal tDCS cerebellar stimulation is more effective in enhancing online motor learning but our results of the current study do not support these findings. They also reported that both the primary motor cortex and the cerebellum play significant roles in the processing of innate motor learning, thereby both enhancing motor learning and this statement was in line with our findings (Ehsani et al., 2016). Michael Doppelmayr et al. also reported in their study that cerebellar stimulation is superior to motor cortex stimulation in facilitating motor adoption (Doppelmayr et al., 2016).

Some studies suggest that tDCS stimulation especially cerebellar stimulation can improve cognition as well or may influence cognition along with motor rehabilitation (Au-Yeung et al., 2014; Ferrucci and Priori, 2014; D’Agata et al., 2016; Draaisma et al., 2020). Keeping in view this fact we also assessed cognitive function using mini-mental state examination and montreal cognitive assessment tool but no significant finding was recorded among any of the three group of interventions.

The majority of stroke patients did not experience any serious adverse effects from tDCS. However, regrettably, many articles that were published did not offer a thorough explanation of exclusion criteria or a comprehensive account of adverse effects (Russo et al., 2017). According to the literature, the most frequent side effect following tDCS is itching followed by tingling, burning, and then headache (Brunoni et al., 2011; Ehsani et al., 2016; Russo et al., 2017) but in the current study contrary to previous studies on tDCS the most common reported adverse effect was headache that was experienced by 72.7% population. None of these subjects asked for the stimulation to stop or require any sort of medical attention during or after the stimulation. A possible reason for the headache might be that the study included patients as Csaba Poreisz et al. and collegues in their study reported higher incidence of headache in patients including stroke, migraine and tinnitus patients in comparison to healthy individuals (Poreisz et al., 2007).

The current study had a few limitations. The fact that majority of the outcome measures had baseline clinical heterogeneity was one of the limitations. Moreover the study investigated effects of short term stimulation only and future studies conducted on long term effects of anodal tDCS may be more helpful in terms of clinical implications. We included stroke patients of all stages, however, acute, sub-acute, and chronic stroke patients may respond to the intervention differently. In addition, a gender effect needs to be further investigated. In the sham group stimulation we used tDCS mounting similar to motor cortex stimulation group only; however using mounting similar to cerebellar group for half of the participants in sham group could be better. It is suggested that future research use more localized electrodes. The current study’s goal was to take both motor and cognitive functions into account and used broader scales that cover different aspects of motor learning. It is also advised that future studies employing specific components of posture, gait parameters, and simple motor tasks should be conducted to compare the effects of cerebellar a-tDCS and M1 in particular.

## Conclusion

In conclusion, the current study suggests short term application of anodal transcranial direct current stimulation on motor cortex and cerebellum along with virtual reality based gait and balance training has promising effects on gait, balance and function of lower extremities in stroke patients. Additionally, the recent research indicates that cerebral cortex stimulation may enhance stroke patients’ cognitive function but future studies should be conducted to separately focus on cognitive function in stroke patients. We will suggest clinicians to add tDCS stimulation as an adjunct therapy with any type of rehabilitation training for stroke patients. Studies assessing long term effect of tDCS stimulation over the two stimulation sites may appear to be more helpful in order to conclude if any of the two stimulation sites for LL and balance function can be declared superior to other.

## Data availability statement

The raw data supporting the conclusion of this article will be made available by the authors from the corresponding author on reasonable request.

## Ethics statement

Research Protocol of the study was approved by the Ethics Committee of Riphah College of Rehabilitation Sciences Riphah International University, Islamabad “Research Ethical Committee” on October 10, 2021 (Ref: RIPHAH/RCRS/REC/Letter/01142). The patients/participants provided their written informed consent to participate in this study.

## Author contributions

Q: conceptualization, analysis, investigation, methodology, writing—original draft, and writing—review and editing. ZA: manuscript writing—review and editing and statistical analysis. SuI: data collection, writing—original draft, and analysis. IS: methodology and statistical analysis and graphical representations. AM: supervision and review and editing. IT: methodology and supervision. TL: funding acquisition, methodology, project administration, and writing—review and editing. JW: conceptualization, funding acquisition, project administration, resources, supervision, and writing—review and editing. All authors contributed to the article and approved the submitted version.

## Footnotes

1
https://www.sealedenvelope.com/

## References

[B1] Andrade S. M. Ferreira J. J. D. A. Rufino T. S. Medeiros G. Brito J. D. Da Silva M. A. (2017). Effects of different montages of transcranial direct current stimulation on the risk of falls and lower limb function after stroke. *Neurol. Res.* 39 1037–1043. 10.1080/01616412.2017.1371473 28885111

[B2] Au-Yeung S. S. Y. Wang J. Chen Y. Chua E. (2014). Transcranial direct current stimulation to primary motor area improves hand dexterity and selective attention in chronic stroke. *Am. J. Phys. Med. Rehabil.* 93 1057–1064. 10.1097/PHM.0000000000000127 24919077

[B3] Baharlouei H. Sadeghi-Demneh E. Mehravar M. Manzari P. Yazdi M. J. S. Joghataei M. T. (2020). Comparison of transcranial direct current stimulation of the primary motor cortex and cerebellum on static balance in older adults. *Iran. Red Crescent. Med. J.* 2.2:e96259. 10.5812/ircmj.96259

[B4] Brunoni A. R. Amadera J. Berbel B. Volz M. S. Rizzerio B. G. Fregni F. J. I. J. O. N. (2011). A systematic review on reporting and assessment of adverse effects associated with transcranial direct current stimulation. *Int. J. Neuropsychopharmacol.* 14 1133–1145. 10.1017/S1461145710001690 21320389

[B5] Caligiore D. Pezzulo G. Baldassarre G. Bostan A. C. Strick P. L. Doya K. (2017). Consensus paper: Towards a systems-level view of cerebellar function: The interplay between cerebellum, basal ganglia, and cortex. *Cerebellum* 16 203–229. 10.1007/s12311-016-0763-3 26873754 PMC5243918

[B6] Celnik P. J. T. C. (2015). Understanding and modulating motor learning with cerebellar stimulation. *Cerebellum* 14 171–174. 10.1007/s12311-014-0607-y 25283180 PMC4348328

[B7] Craig C. E. Doumas M. J. P. O. (2017). Anodal transcranial direct current stimulation shows minimal, measure-specific effects on dynamic postural control in young and older adults: A double blind, sham-controlled study. *PLoS One* 12:e0170331. 10.1371/journal.pone.0170331 28099522 PMC5242524

[B8] D’Agata F. Peila E. Cicerale A. Caglio M. M. Caroppo P. Vighetti S. (2016). Cognitive and neurophysiological effects of non-invasive brain stimulation in stroke patients after motor rehabilitation. *Front. Behav. Neurosci.* 10:135. 10.3389/fnbeh.2016.00135 27445730 PMC4919333

[B9] Doppelmayr M. Pixa N. H. Steinberg F. J. J. O. T. I. N. S. (2016). Cerebellar, but not motor or parietal, high-density anodal transcranial direct current stimulation facilitates motor adaptation. *J. Int. Neuropsychol. Soc.* 22 928–936. 10.1017/S1355617716000345 27152869

[B10] Doya K. J. C. O. I. N. (2000). Complementary roles of basal ganglia and cerebellum in learning and motor control. *Curr. Opin. Neurobiol.* 10 732–739. 10.1016/S0959-4388(00)00153-7 11240282

[B11] Draaisma L. R. Wessel M. J. Hummel F. C. J. N. L. (2020). Non-invasive brain stimulation to enhance cognitive rehabilitation after stroke. *Curr. Opin. Neurobiol.* 719:133678. 10.1016/j.neulet.2018.06.047 29960054

[B12] Ehsani F. Bakhtiary A. Jaberzadeh S. Talimkhani A. Hajihasani A. J. N. R. (2016). Differential effects of primary motor cortex and cerebellar transcranial direct current stimulation on motor learning in healthy individuals: A randomized double-blind sham-controlled study. *Neurosci. Res.* 112 10–19. 10.1016/j.neures.2016.06.003 27349154

[B13] Ehsani F. Mortezanejad M. Yosephi M. H. Daniali S. Jaberzadeh S. (2022). The effects of concurrent M1 anodal tdcs and physical therapy interventions on function of ankle muscles in patients with stroke: A randomized, double-blinded sham-controlled trial study. *Neurol. Sci.* 43 1893–1901. 10.1007/s10072-021-05503-9 34476629

[B14] Faul F. Erdfelder E. Lang A.-G. Buchner A. J. B. R. M. (2007). G* Power 3: A flexible statistical power analysis program for the social, behavioral, and biomedical sciences. *Behav. Res. Methods* 39 175–191. 10.3758/BF03193146 17695343

[B15] Ferrucci R. Priori A. J. N. (2014). Transcranial cerebellar direct current stimulation (tcdcs): Motor control, cognition, learning and emotions. *Neuroimage* 85 918–923. 10.1016/j.neuroimage.2013.04.122 23664951

[B16] Ferrucci R. Brunoni A. R. Parazzini M. Vergari M. Rossi E. Fumagalli M. (2013). Modulating human procedural learning by cerebellar transcranial direct current stimulation. *Cerebellum* 12 485–492. 10.1007/s12311-012-0436-9 23328908

[B17] Foerster Á Melo L. Mello M. Castro R. Shirahige L. Rocha S. (2017). Cerebellar transcranial direct current stimulation (ctdcs) impairs balance control in healthy individuals. *Cerebellum* 16 872–875. 10.1007/s12311-017-0863-8 28456902

[B18] Galea J. M. Jayaram G. Ajagbe L. Celnik P. J. J. O. N. (2009). Modulation of cerebellar excitability by polarity-specific noninvasive direct current stimulation. *J. Neurosci.* 29 9115–9122. 10.1523/JNEUROSCI.2184-09.2009 19605648 PMC2760225

[B19] Galea J. M. Vazquez A. Pasricha N. Orban, De Xivry J.-J. Celnik P. J. C. C. (2011). Dissociating the roles of the cerebellum and motor cortex during adaptive learning: The motor cortex retains what the cerebellum learns. *Cereb. Cortex* 21 1761–1770. 10.1093/cercor/bhq246 21139077 PMC3138512

[B20] Gandiga P. C. Hummel F. C. Cohen L. G. J. C. N. (2006). Transcranial Dc stimulation (tdcs): A tool for double-blind sham-controlled clinical studies in brain stimulation. *Clin. Neurophysiol.* 117 845–850. 10.1016/j.clinph.2005.12.003 16427357

[B21] Geroin C. Picelli A. Munari D. Waldner A. Tomelleri C. Smania N. J. C. R. (2011). Combined transcranial direct current stimulation and robot-assisted gait training in patients with chronic stroke: A preliminary comparison. *Clin. Rehabil.* 25 537–548. 10.1177/0269215510389497 21402651

[B22] Gowan S. Hordacre B. J. B. S. (2020). Transcranial direct current stimulation to facilitate lower limb recovery following stroke: Current evidence and future directions. *Brain Sci.* 10:310. 10.3390/brainsci10050310 32455671 PMC7287858

[B23] Hardwick R. M. Celnik P. A. J. N. O. A. (2014). Cerebellar direct current stimulation enhances motor learning in older adults. *Neurobiol. Aging* 35 2217–2221. 10.1016/j.neurobiolaging.2014.03.030 24792908 PMC4087063

[B24] Hummel F. Celnik P. Giraux P. Floel A. Wu W.-H. Gerloff C. (2005). Effects of non-invasive cortical stimulation on skilled motor function in chronic stroke. *Brain* 128 490–499. 10.1093/brain/awh369 15634731

[B25] Ito M. Yamaguchi K. Nagao S. Yamazaki T. J. P. I. B. R. (2014). Long-term depression as a model of cerebellar plasticity. *Prog. Brain Res.* 210 1–30. 10.1016/B978-0-444-63356-9.00001-7 24916287

[B26] Jayaram G. Galea J. M. Bastian A. J. Celnik P. J. C. C. (2011). Human locomotor adaptive learning is proportional to depression of cerebellar excitability. *Cereb. Cortex* 21 1901–1909. 10.1093/cercor/bhq263 21239392 PMC3202738

[B27] Jeffery D. T. Norton J. A. Roy F. D. Gorassini M. A. J. E. B. R. (2007). Effects of transcranial direct current stimulation on the excitability of the leg motor cortex. *Exp. Brain Res.* 182 281–287. 10.1007/s00221-007-1093-y 17717651

[B28] Jung S. Song S. Lee D. Lee K. Lee G. J. D. N. (2021). Effects of kinect video game training on lower extremity motor function, balance, and gait in adolescents with spastic diplegia cerebral palsy: A pilot randomized controlled trial. *Dev. Neurorehabil.* 24 159–165. 10.1080/17518423.2020.1819458 32981401

[B29] Jung S.-H. Song S.-H. Kim S.-D. Lee K. Lee G.-C. J. J. O. P. R. M. (2018). Does virtual reality training using the Xbox Kinect have a positive effect on physical functioning in children with spastic cerebral palsy? A case series. *J. Pediatr. Rehabil. Med.* 11 95–101. 10.3233/PRM-160415 30010148

[B30] Kaski D. Quadir S. Patel M. Yousif N. Bronstein A. M. J. J. O. N. (2012). Enhanced locomotor adaptation aftereffect in the “broken escalator” phenomenon using anodal tdcs. *J. Neurophysiol.* 107 2493–2505. 10.1152/jn.00223.2011 22323638 PMC3362242

[B31] Kim C. R. Kim D.-Y. Kim L. S. Chun M. H. Kim S. J. Park C. H. J. B. S. (2012). Modulation of cortical activity after anodal transcranial direct current stimulation of the lower limb motor cortex: A functional Mri study. *Brain Stimul.* 5 462–467. 10.1016/j.brs.2011.08.002 21962977

[B32] Kim H. Lee G. Song C. J. J. O. S. Diseases C. (2014). Effect of functional electrical stimulation with mirror therapy on upper extremity motor function in poststroke patients. *J. Stroke Cerebrovasc. Dis.* 23 655–661. 10.1016/j.jstrokecerebrovasdis.2013.06.017 23867040

[B33] Krings T. Töpper R. Foltys H. Erberich S. Sparing R. Willmes K. (2000). Cortical activation patterns during complex motor tasks in piano players and control subjects. A functional magnetic resonance imaging study. *Neurosci. Lett.* 278 189–193. 10.1016/S0304-3940(99)00930-1 10653025

[B34] Langhorne P. Coupar F. Pollock A. J. T. L. N. (2009). Motor recovery after stroke: A systematic review. *Lancet Neurol.* 8 741–754. 10.1016/S1474-4422(09)70150-4 19608100

[B35] Liebrand M. Karabanov A. Antonenko D. Flöel A. Siebner H. R. Classen J. (2020). Beneficial effects of cerebellar tdcs on motor learning are associated with altered putamen-cerebellar connectivity: A simultaneous tdcs-fmri study. *NeuroImage* 223:117363. 10.1016/j.neuroimage.2020.117363 32919057

[B36] Luna-Oliva L. Ortiz-Gutiérrez R. M. Cano-De, La Cuerda R. Piédrola R. M. Alguacil-Diego I. M. (2013). Kinect Xbox 360 as a therapeutic modality for children with cerebral palsy in a school environment: A preliminary study. *NeuroRehabilitation* 33 513–521. 10.3233/NRE-131001 24018364

[B37] MacLullich A. M. Edmond C. L. Ferguson K. J. Wardlaw J. M. Starr J. M. Seckl J. R. (2004). Size of the neocerebellar vermis is associated with cognition in healthy elderly men. *Brain Cogn.* 56 344–348. 10.1016/j.bandc.2004.08.001 15522773

[B38] Manji A. Amimoto K. Matsuda T. Wada Y. Inaba A. Ko S. J. N. L. (2018). Effects of transcranial direct current stimulation over the supplementary motor area body weight-supported treadmill gait training in hemiparetic patients after stroke. *Neurosci. Lett.* 662 302–305. 10.1016/j.neulet.2017.10.049 29107706

[B39] Mohammadi R. Mahmoudi Z. Mahmoodian N. J. M. E. J. O. R. Studies H. (2021). Effects of cerebellar transcranial direct current stimulation (tdcs) on timed up and go test with foot placement in chronic stroke patients. *Middle East J. Rehabil. Health Stud.* 8:e106180. 10.5812/mejrh.106180

[B40] Morton S. M. Bastian A. J. J. T. N. (2004). Cerebellar control of balance and locomotion. *Neuroscientist* 10 247–259. 10.1177/1073858404263517 15155063

[B41] Navarro-López V. Molina-Rueda F. Jiménez-Jiménez S. Alguacil-Diego I. M. Carratalá-Tejada M. J. D. (2021). Effects of transcranial direct current stimulation combined with physiotherapy on gait pattern, balance, and functionality in stroke patients. A systematic review. *Diagnostics (Basel)* 11:656. 10.3390/diagnostics11040656 33916442 PMC8066876

[B42] Nitsche M. A. Paulus W. J. N. (2001). Sustained excitability elevations induced by transcranial Dc motor cortex stimulation in humans. *Neurology* 57 1899–1901. 10.1212/WNL.57.10.1899 11723286

[B43] Nitsche M. A. Paulus W. J. T. J. O. P. (2000). Excitability changes induced in the human motor cortex by weak transcranial direct current stimulation. *J. Physiol.* 527:633. 10.1111/j.1469-7793.2000.t01-1-00633.x 10990547 PMC2270099

[B44] Nitsche M. A. Liebetanz D. Lang N. Antal A. Tergau F. Paulus W. J. C. N. O. J. O. T. I. F. O. C. N. (2003). Safety criteria for transcranial direct current stimulation (tdcs) in humans. *Clin. Neurophysiol.* 114 2220–2223. 10.1016/S1388-2457(03)00235-9 14580622

[B45] Norrving B. Mensah G. J. C. R. (2017). Global burden of stroke. *Semin. Neurol.* 120 439–448. 10.1161/CIRCRESAHA.116.308413 28154096

[B46] Paul R. Grieve S. M. Chaudary B. Gordon N. Lawrence J. Cooper N. (2009). Relative contributions of the cerebellar vermis and prefrontal lobe volumes on cognitive function across the adult lifespan. *Neurobiol. Aging* 30 457–465. 10.1016/j.neurobiolaging.2007.07.017 17869383

[B47] Picelli A. Chemello E. Castellazzi P. Filippetti M. Brugnera A. Gandolfi M. (2018). Combined effects of cerebellar transcranial direct current stimulation and transcutaneous spinal direct current stimulation on robot-assisted gait training in patients with chronic brain stroke: A pilot, single blind, randomized controlled trial. *Restor. Neurol. Neurosci.* 36 161–171. 10.3233/RNN-170784 29526857

[B48] Poortvliet P. Hsieh B. Cresswell A. Au J. Meinzer M. J. C. N. (2018). . Cerebellar transcranial direct current stimulation improves adaptive postural control. *Clin. Neurophysiol.* 129 33–41. 10.1016/j.clinph.2017.09.118 29136550

[B49] Poreisz C. Boros K. Antal A. Paulus W. J. B. R. B. (2007). Safety aspects of transcranial direct current stimulation concerning healthy subjects and patients. *Brain Res. Bull.* 72 208–214. 10.1016/j.brainresbull.2007.01.004 17452283

[B50] Rampersad S. M. Janssen A. M. Lucka F. Aydin Ü Lanfer B. Lew S. (2014). Simulating transcranial direct current stimulation with a detailed anisotropic human head model. *IEEE Trans. Neural. Syst. Rehabil. Eng.* 22 441–452. 10.1109/TNSRE.2014.2308997 24760939

[B51] Reis J. Schambra H. M. Cohen L. G. Buch E. R. Fritsch B. Zarahn E. (2009). Noninvasive cortical stimulation enhances motor skill acquisition over multiple days through an effect on consolidation. *Proc. Natl. Acad. Sci. U.S.A.* 106 1590–1595. 10.1073/pnas.0805413106 19164589 PMC2635787

[B52] Rezaee Z. Ranjan S. Solanki D. Bhattacharya M. Srivastava M. Lahiri U. (2021). Feasibility of combining functional near-infrared spectroscopy with electroencephalography to identify chronic stroke responders to cerebellar transcranial direct current stimulation—A computational modeling and portable neuroimaging methodological study. *Cerebellum* 20 853–871. 10.1007/s12311-021-01249-4 33675516

[B53] Richards C. L. Malouin F. Dean C. J. C. I. G. M. (1999). Gait in stroke: Assessment and rehabilitation. *Clin. Geriatr. Med.* 15 833–856. 10.1016/S0749-0690(18)30034-X 10499938

[B54] Robinson C. A. Shumway-Cook A. Matsuda P. N. Ciol M. A. J. D. Rehabilitation. (2011). Understanding physical factors associated with participation in community ambulation following stroke. *Disabil. Rehabil.* 33 1033–1042. 10.3109/09638288.2010.520803 20923316

[B55] Roche N. Geiger M. Bussel B. J. A. O. P. Medicine R. (2015). Mechanisms underlying transcranial direct current stimulation in rehabilitation. *Ann. Phys. Rehabil. Med.* 58 214–219. 10.1016/j.rehab.2015.04.009 26189791

[B56] Roche N. Lackmy A. Achache V. Bussel B. Katz R. J. T. J. O. P. (2009). Impact of transcranial direct current stimulation on spinal network excitability in humans. *J. Physiol.* 587 5653–5664. 10.1113/jphysiol.2009.177550 19805746 PMC2805376

[B57] Russo C. Souza Carneiro M. I. Bolognini N. Fregni F. (2017). Safety review of transcranial direct current stimulation in stroke. *Neuromodulation* 20 215–222. 10.1111/ner.12574 28220641 PMC5389927

[B58] Salameh A. Mccabe J. Skelly M. Duncan K. R. Chen Z. Tatsuoka C. (2022). Stance phase gait training post stroke using simultaneous transcranial direct current stimulation and motor learning-based virtual reality-assisted therapy: Protocol development and initial testing. *Brain Sci.* 12:701. 10.3390/brainsci12060701 35741586 PMC9221094

[B59] Samaei A. Ehsani F. Zoghi M. Hafez Yosephi M. Jaberzadeh S. J. E. J. O. N. (2017). Online and offline effects of cerebellar transcranial direct current stimulation on motor learning in healthy older adults: A randomized double-blind sham-controlled study. *Eur. J. Neurosci.* 45 1177–1185. 10.1111/ejn.13559 28278354

[B60] Schlerf J. E. Galea J. M. Spampinato D. Celnik P. A. J. C. C. (2015). Laterality differences in cerebellar–motor cortex connectivity. *Cereb. Cortex* 25 1827–1834. 10.1093/cercor/bht422 24436320 PMC4459286

[B61] Seo H. G. Lee W. H. Lee S. H. Yi Y. Kim K. D. Oh B.-M. (2017). Robotic-assisted gait training combined with transcranial direct current stimulation in chronic stroke patients: A pilot double-blind, randomized controlled trial. *Restor. Neurol. Neurosci.* 35 527–536. 10.3233/RNN-170745 28800341

[B62] Shumway-Cook A. Woollacott M. H. (2007). *Motor control: Translating research into clinical practice.* Philadelphia: Lippincott Williams & Wilkins.

[B63] Sohn M. K. Jee S. J. Kim Y. W. J. A. O. R. M. (2013). Effect of transcranial direct current stimulation on postural stability and lower extremity strength in hemiplegic stroke patients. *Ann. Rehabil. Med.* 37 759–765. 10.5535/arm.2013.37.6.759 24466510 PMC3895515

[B64] Solanki D. Rezaee Z. Dutta A. Lahiri U. J. J. O. N. Rehabilitation. (2021). Investigating the feasibility of cerebellar transcranial direct current stimulation to facilitate post-stroke overground gait performance in chronic stroke: A partial least-squares regression approach. *J. Neuroeng. Rehabil.* 18 1–18. 10.1186/s12984-021-00817-3 33509192 PMC7842063

[B65] Stagg C. J. Nitsche M. A. J. T. N. (2011). Physiological basis of transcranial direct current stimulation. *Neuroscientist* 17 37–53. 10.1177/1073858410386614 21343407

[B66] Stoykov M. E. Madhavan S. J. J. O. N. P. T. J. (2015). Motor priming in neurorehabilitation. *J. Neurol. Phys. Ther.* 39:33. 10.1097/NPT.0000000000000065 25415551 PMC4270918

[B67] Ungerleider L. G. Doyon J. Karni A. J. N. O. L. Memory. (2002). Imaging brain plasticity during motor skill learning. *Neurobiol. Learn. Mem.* 78 553–564. 10.1006/nlme.2002.4091 12559834

[B68] Vaz P. G. Salazar A. P. D. S. Stein C. Marchese R. R. Lukrafka J. L. Plentz R. D. M. (2019). Noninvasive brain stimulation combined with other therapies improves gait speed after stroke: A systematic review and meta-analysis. *Top Stroke Rehabil.* 26 201–213. 10.1080/10749357.2019.1565696 30735104

[B69] Vines B. W. Nair D. Schlaug G. J. E. J. O. N. (2008). Modulating activity in the motor cortex affects performance for the two hands differently depending upon which hemisphere is stimulated. *Eur. J. Neurosci.* 28 1667–1673. 10.1111/j.1460-9568.2008.06459.x 18973584

[B70] Xu Y. Hou Q. H. Russell S. D. Bennett B. C. Sellers A. J. Lin Q. (2015). Neuroplasticity in post-stroke gait recovery and noninvasive brain stimulation. *Neural Regen. Res.* 10:2072–2080. 10.4103/1673-5374.172329 26889202 PMC4730838

[B71] Yosephi M. H. Ehsani F. Zoghi M. Jaberzadeh S. J. B. S. (2018). Multi-session anodal tdcs enhances the effects of postural training on balance and postural stability in older adults with high fall risk: Primary motor cortex versus cerebellar stimulation. *Brain Stimul.* 11 1239–1250. 10.1016/j.brs.2018.07.044 30017699

[B72] Zandvliet S. B. Meskers C. G. Kwakkel G. Van Wegen E. E. J. T. C. (2018). Short-term effects of cerebellar tdcs on standing balance performance in patients with chronic stroke and healthy age-matched elderly. *Cerebellum* 17 575–589. 10.1007/s12311-018-0939-0 29797226 PMC6132826

[B73] Zuchowski M. L. Timmann D. Gerwig M. J. B. S. (2014). Acquisition of conditioned eyeblink responses is modulated by cerebellar tdcs. *Brain Stimul.* 7 525–531. 24776785 10.1016/j.brs.2014.03.010

