# Supplementary material for: Short term effects of anodal cerebellar vs. anodal cerebral transcranial direct current stimulation in stroke patients, a randomized control trial
Source: Front Neurosci. 2022 Nov 24;16:1035558. doi: 10.3389/fnins.2022.1035558 (PMC9730515; doi:10.3389/fnins.2022.1035558)
Supplement: Supplementary file 1 [file Table_1.DOCX]

Table - Across Group Analysis for normally distributed variables using Mixed/Split Plot ANOVA

| **Variables** | **Cerebellar Stimulation Group** | **M1 Stimulation Group** | **Sham Stimulation Group** | | **P- Value** | **df** | **Effect Size** |
| --- | --- | --- | --- | --- | --- | --- | --- |
|  | **CbSG (Mean ± SD)** | **MSG (Mean ± SD)** | | **SSG (Mean ± SD)** |  |  | **Partial Eta Squared** |
| Montreal Cognitive Assessment Pre | 17.73 ± 4.9 | 18.09 ± 4.7 | 18.73 ± 4.0 | | 0.941 | 2 |  |
| Montreal Cognitive Assessment Post | 23.82 ± 2.3 | 23.36 ± 2.9 | 23.36 ± 2.7 | |  |  |  |
| Mean Difference | 6.10 ± 3.3 | 5.27 ± 4.5 | 4.64 ± 2.4 | | 0.396 | 2 | 0.029 |
| BESTest Reactive Postural Response Pre | 8.82 ± 2.3 | 7.18 ± 3.2 | 9.90 ± 2.7 | | 0.008* | 2 |  |
| BESTest Reactive Postural Response Post | 13.18 ± 2.4 | 11.36 ± 2.0 | 13.18 ± 2.5 | |  |  |  |
| Mean Difference | 4.36 ± 1.5 | 4.18 ± 1.8 | 3.27 ± 1.1 | | 0.041* | 2 | 0.096 |
| BESTest Stability In Gait Pre | 9.64 ± 5.8 | 9.45± 4.5 | 14.36 ± 2.5 | | 0.001* | 2 |  |
| BESTest Stability In Gait Post | 14.09 ± 4.0 | 14.73 ± 2.7 | 16.91 ± 2.7 | |  |  |  |
| Mean Difference | 4.45 ± 3.8 | 5.27 ± 4.6 | 2.55 ± 1.9 | | 0.045* | 2 | 0.094 |
