# Supplementary material for: Short term effects of anodal cerebellar vs. anodal cerebral transcranial direct current stimulation in stroke patients, a randomized control trial
Source: Front Neurosci. 2022 Nov 24;16:1035558. doi: 10.3389/fnins.2022.1035558 (PMC9730515; doi:10.3389/fnins.2022.1035558)
Supplement: Supplementary file 2 [file Table_2.DOCX]

Table - Across Group Analysis for skewed variables using Kruskal-Wallis H Test

| **Variables** | **Cerebellar Stimulation Group** | **M1 Stimulation Group** | **Sham Stimulation Group** | **P- Value** | **df** | **H** | **Effect Size** |
| --- | --- | --- | --- | --- | --- | --- | --- |
|  | **CbSG (Mean ± SD)** | **MSG (Mean ± SD)** | **SSG (Mean ± SD)** |  |  |  | **Partial Eta Squared** |
| BBS Pre | 41.63 ± 8.2 | 40.27 ± 8.4 | 50.27 ± 3.0 | 0.001 | 2 | 19.54 |  |
| BBS Post | 47.91 ± 7.7 | 49.36 ± 3.6 | 52.36 ± 3.0 | 0.047 | 2 | 6.13 |  |
| BBS Mean Difference | 6.27 ± 4.4 | 9.09 ± 9.01 | 2.09 ± 1.3 | 0.001* | 2 | 14.10 | 0.2 |
| TUG Pre | 17.55 ± 7.6 | 13.86 ± 4.9 | 9.69 ± 2.9 | 0.001 | 2 | 17.36 |  |
| TUG Post | 12.51 ± 6.3 | 10.58 ± 4.6 | 7.99 ± 2.4 | 0.012 | 2 | 8.84 |  |
| TUG Mean Difference | 5.03 ± 4.2 | 3.28 ± 3.1 | 1.71 ± 1.5 | 0.004* | 2 | 11.01 | 0.163 |
| 6MWT Pre | 0.12 ± 0.03 | 0.13 ± 0.03 | 0.17 ± 0.08 | 0.013 | 2 | 6.75 |  |
| 6MWT Post | 0.18 ± 0.04 | 0.17 ± 0.05 | 0.23 ± 0.09 | 0.034 | 2 | 17.36 |  |
| 6MWT Mean Difference | 0.06 ± 0.03 | 0.04 ± 0.03 | 0.06 ± 0.05 | 0.435 | 2 | 1.66 | 0.048 |
| 25FWT Pre | 18.47 ± 12.8 | 14.07 ± 6.8 | 10.60 ± 7.01 | 0.01 | 2 | 9.21 |  |
| 25FWT Post | 14.31 ± 10.1 | 11.95 ± 6.7 | 8.02 ± 2.9 | 0.023 | 2 | 7.51 |  |
| 25FWT Mean Difference | 4.15 ± 3.9 | 2.21 ± 2.1 | 2.58 ± 2.4 | 0.141 | 2 | 3.92 | 0.039 |
| JHFRAT Pre | 9.82 ± 4.9 | 12.82 ± 3.5 | 10.00 ± 2.8 | 0.026 | 2 | 7.31 |  |
| JHFRAT Post | 12.18 ± 2.7 | 12.81 ± 3.5 | 10.00 ± 2.7 | 0.02 | 2 | 7.81 |  |
| JHFRAT Mean Difference | 2.36 ± 5.1 | 0.00 ± 0.0 | 0.00 ± 0.0 | 0.015* | 2 | 8.39 | 0.129 |
| BESTest Total Pre | 62.73 ± 15.3 | 59.27 ± 17.7 | 75.18 ± 10.1 | 0.015 | 2 | 8.44 |  |
| BESTest Total Post | 77.81 ± 9.9 | 77.36 ± 9.2 | 84.09 ± 9.6 | 0.055 | 2 | 5.81 |  |
| BESTest Total Mean Difference | 15.09 ± 9.3 | 18.09 ± 13.2 | 8.91 ± 4.2 | 0.004* | 2 | 10.85 | 0.142 |
| BESTest subgroup 1 Pre | 8.45 ± 2.5 | 7.54 ± 3.9 | 10.00 ± 2.3 | 0.102 | 2 | 4.57 |  |
| BESTest subgroup 1 Post | 9.72 ± 1.2 | 9.36 ± 3.3 | 10.45 ± 2.6 | 0.555 | 2 | 1.18 |  |
| BESTest subgroup 1 Mean Difference | 1.27 ± 2.1 | 1.82 ± 2.1 | 0.45 ± 0.8 | 0.034* | 2 | 6.79 | 0.092 |
| BESTest subgroup 2 Pre | 13.54 ± 3.9 | 14.09 ± 3.55 | 15.36 ± 1.9 | 0.388 | 2 | 1.89 |  |
| BESTest subgroup 2 Post | 15.91 ± 2.2 | 16.45 ± 1.9 | 16.36 ± 1.8 | 0.851 | 2 | 0.32 |  |
| BESTest subgroup 2 Mean Difference | 2.36 ± 2.3 | 2.36 ± 2.7 | 1.00 ± 0.7 | 0.215 | 2 | 3.07 | 0.087 |
| BESTest subgroup 3 Pre | 8.36 ± 2.3 | 8.54 ± 2.9 | 11.54 ± 2.8 | 0.001 | 2 | 13.92 |  |
| BESTest subgroup 3 Post | 10.54 ± 1.9 | 11.18 ± 2.2 | 12.63 ± 2.6 | 0.025 | 2 | 7.34 |  |
| BESTest subgroup 3 Mean Difference | 2.18 ± 1.3 | 2.64 ± 1.8 | 1.09 ± 1.1 | 0.004* | 2 | 11.15 | 0.153 |
| BESTest subgroup 5 Pre | 12.72 ± 2.3 | 12.63 ± 2.2 | 14.00 ± 0.8 | 0.044 | 2 | 6.26 |  |
| BESTest subgroup 5 Post | 14.36 ± 0.9 | 14.27 ± 0.6 | 14.54 ± 0.5 | 0.354 | 2 | 2.08 |  |
| BESTest subgroup 5 Mean Difference | 1.64 ± 2.0 | 1.64 ± 2.2 | 0.55 ± 0.6 | 0.074 | 2 | 5.19 | 0.08 |
| MMSE pre | 22.82 ± 4.5 | 20.73 ± 5.0 | 23.45 ± 5.7 | 0.1 | 2 | 4.74 |  |
| MMSE Post | 25.55 ± 3.3 | 25.18 ± 3.2 | 25.63 ± 3.7 | 0.655 | 2 | 0.85 |  |
| MMSE Mean Difference | 2.73 ± 2.1 | 4.45 ± 3.1 | 2.18 ± 2.1 | 0.027* | 2 | 7.25 | 0.128 |
