# Supplementary material for: Short term effects of anodal cerebellar vs. anodal cerebral transcranial direct current stimulation in stroke patients, a randomized control trial
Source: Front Neurosci. 2022 Nov 24;16:1035558. doi: 10.3389/fnins.2022.1035558 (PMC9730515; doi:10.3389/fnins.2022.1035558)
Supplement: Supplementary file 3 [file Table_3.DOCX]

Table - Bonferroni’s method for Pair wise Multiple Comparisons

| **Variable** | **Pairwise Comparison** | **Mean Difference (95% CI)** | **P- Value** |
| --- | --- | --- | --- |
|  |  |  |  |
| Berg Balance Scale | Cerebellar Stimulation Group (CbSG) | 2.82 (1.54 -7.18) | 0.199 |
|  | M1 Stimulation Group (MSG) |  |  |
|  | Cerebellar Stimulation Group (CbSG) | 4.18 (2.19 - 6.17) | **<0.001*** |
|  | Sham Stimulation Group (SSG) |  |  |
|  | M1 Stimulation Group (MSG) | 7.00 (3.04 -10.95) | **0.001*** |
|  | Sham Stimulation Group (SSG) |  |  |
| Timed Up and Go Test | Cerebellar Stimulation Group (CbSG) | 1.75 (0.49 - 3.99) | 0.123 |
|  | M1 Stimulation Group (MSG) |  |  |
|  | Cerebellar Stimulation Group (CbSG) | 3.33 (1.39 - 5.26) | **0.001*** |
|  | Sham Stimulation Group (SSG) |  |  |
|  | M1 Stimulation Group (MSG) | 1.58 (0.07 - 3.08) | **0.041*** |
|  | Sham Stimulation Group (SSG) |  |  |
| Six Minute Walk Test | Cerebellar Stimulation Group (CbSG) | 0.012 (0.01 - 0.04) | 0.123 |
|  | M1 Stimulation Group (MSG) |  |  |
|  | Cerebellar Stimulation Group (CbSG) | 0.006 (0.02 - 0.03) | 0.641 |
|  | Sham Stimulation Group (SSG) |  |  |
|  | M1 Stimulation Group (MSG) | 0.023 (0.01 - 0.05) | 0.107 |
|  | Sham Stimulation Group (SSG) |  |  |
| 25 Feet Walk Test | Cerebellar Stimulation Group (CbSG) | 2.04 (0.71 - 4.78) | 0.142 |
|  | M1 Stimulation Group (MSG) |  |  |
|  | Cerebellar Stimulation Group (CbSG) | 1.57 (1.57 - 4.71) | 0.318 |
|  | Sham Stimulation Group (SSG) |  |  |
|  | M1 Stimulation Group (MSG) | 0.465 (1.62-2.55) | 0.655 |
|  | Sham Stimulation Group (SSG) |  |  |
| Johns Hopkins Fall Risk Assessment Tool | Cerebellar Stimulation Group (CbSG) | 2.36 (0.16 - 4.57) | **0.037*** |
|  | M1 Stimulation Group (MSG) |  |  |
|  | Cerebellar Stimulation Group (CbSG) | 2.36 (0.16 - 4.57) | **0.037*** |
|  | Sham Stimulation Group (SSG) |  |  |
|  | M1 Stimulation Group (MSG) | 0.00 (0.0 - 0.0) | 0 |
|  | Sham Stimulation Group (SSG) |  |  |
| BESTest Balance Evaluation – Systems Test | Cerebellar Stimulation Group (CbSG) | 3.00 (3.94 - 9.94) | 0.388 |
|  | M1 Stimulation Group (MSG) |  |  |
|  | Cerebellar Stimulation Group (CbSG) | 6.18 (1.79 - 10.56) | **0.007*** |
|  | Sham Stimulation Group (SSG) |  |  |
|  | M1 Stimulation Group (MSG) | 9.18 (3.23 - 15.13) | **0.003*** |
|  | Sham Stimulation Group (SSG) |  |  |
| BESTest Biomechanical Constraints | Cerebellar Stimulation Group (CbSG) | 0.55 (0.75 - 1.85) | 0.402 |
|  | M1 Stimulation Group (MSG) |  |  |
|  | Cerebellar Stimulation Group (CbSG) | 0.82 (0.17 - 1.80) | 0.101 |
|  | Sham Stimulation Group (SSG) |  |  |
|  | M1 Stimulation Group (MSG) | 1.36 (0.39 - 2.34) | **0.007*** |
|  | Sham Stimulation Group (SSG) |  |  |
| BESTest Stability Limits | Cerebellar Stimulation Group (CbSG) | 0.00 (1.55 - 1.55) | 1 |
|  | M1 Stimulation Group (MSG) |  |  |
|  | Cerebellar Stimulation Group (CbSG) | 1.36 (0.29 - 2.43) | **0.013*** |
|  | Sham Stimulation Group (SSG) |  |  |
|  | M1 Stimulation Group (MSG) | 1.36 (0.14 - 2.59) | **0.029*** |
|  | Sham Stimulation Group (SSG) |  |  |
| BESTest Transitions-Anticipatory Postural Adjustment | Cerebellar Stimulation Group (CbSG) | 0.46 (0.51 - 1.41) | 0.344 |
|  | M1 Stimulation Group (MSG) |  |  |
|  | Cerebellar Stimulation Group (CbSG) | 1.09 (0.23 - 1.96) | **0.015*** |
|  | Sham Stimulation Group (SSG) |  |  |
|  | M1 Stimulation Group (MSG) | 1.55 (0.52 - 2.57) | **0.004*** |
|  | Sham Stimulation Group (SSG) |  |  |
| BESTest Reactive Postural Response | Cerebellar Stimulation Group (CbSG) | 0.18 (0.83 - 1.19) | 0.719 |
|  | M1 Stimulation Group (MSG) |  |  |
|  | Cerebellar Stimulation Group (CbSG) | 1.09 (0.29 - 1.89) | **0.009*** |
|  | Sham Stimulation Group (SSG) |  |  |
|  | M1 Stimulation Group (MSG) | 0.91 (0.01 - 1.81) | **0.048*** |
|  | Sham Stimulation Group (SSG) |  |  |
| BESTest Sensory Orientation | Cerebellar Stimulation Group (CbSG) | 0.00 (1.29 - 1.29) | 1 |
|  | M1 Stimulation Group (MSG) |  |  |
|  | Cerebellar Stimulation Group (CbSG) | 1.09 (0.16 - 2.02) | **0.023*** |
|  | Sham Stimulation Group (SSG) |  |  |
|  | M1 Stimulation Group (MSG) | 1.09 (0.10 - 2.08) | **0.031*** |
|  | Sham Stimulation Group (SSG) |  |  |
| BESTest Stability In Gait | Cerebellar Stimulation Group (CbSG) | 0.82 (1.75 - 3.39) | 0.524 |
|  | M1 Stimulation Group (MSG) |  |  |
|  | Cerebellar Stimulation Group (CbSG) | 1.91 (0.07 - 3.75) | 0.91 |
|  | Sham Stimulation Group (SSG) |  |  |
|  | M1 Stimulation Group (MSG) | 2.73 (0.57 - 4.89) | **0.015*** |
|  | Sham Stimulation Group (SSG) |  |  |
| Mini-Mental State Examination | Cerebellar Stimulation Group (CbSG) | 1.73 (0.12 - 3.34) | **0.036*** |
|  | M1 Stimulation Group (MSG) |  |  |
|  | Cerebellar Stimulation Group (CbSG) | 0.55 (0.83 - 1.92) | 0.429 |
|  | Sham Stimulation Group (SSG) |  |  |
|  | M1 Stimulation Group (MSG) | 2.27 (0.56 - 3.99) | **0.011*** |
|  | Sham Stimulation Group (SSG) |  |  |
| Montreal Cognitive Assessment | Cerebellar Stimulation Group (CbSG) | 0.82 (1.59 - 3.23) | 0.497 |
|  | M1 Stimulation Group (MSG) |  |  |
|  | Cerebellar Stimulation Group (CbSG) | 1.46 (0.30 - 3.21) | 0.103 |
|  | Sham Stimulation Group (SSG) |  |  |
|  | M1 Stimulation Group (MSG) | 0.64 (1.58 - 2.85) | 0.565 |
|  | Sham Stimulation Group (SSG) |  |  |
